# Resisting death by metal: metabolism and Cu/Zn homeostasis in bacteria

**DOI:** 10.1042/ETLS20230115

**Published:** 2024-02-16

**Authors:** Matthew J. Sullivan, Ignacio Terán, Kelvin G.K. Goh, Glen C. Ulett

**Affiliations:** 1 School of Biological Sciences, University of East Anglia, Norwich NR4 7TJ, U.K.; 2 School of Pharmacy and Medical Sciences, and Menzies Health Institute Queensland, Griffith University, Gold Coast Campus, Gold Coast, QLD 4222, Australia

**Keywords:** copper, homeostasis, metabolism, metal, small molecules, zinc

## Abstract

Metal ions such as zinc and copper play important roles in host–microbe interactions and their availability can drastically affect the survival of pathogenic bacteria in a host niche. Mechanisms of metal homeostasis protect bacteria from starvation, or intoxication, defined as when metals are limiting, or in excess, respectively. In this mini-review, we summarise current knowledge on the mechanisms of resistance to metal stress in bacteria, focussing specifically on the homeostasis of cellular copper and zinc. This includes a summary of the factors that subvert metal stress in bacteria, which are independent of metal efflux systems, and commentary on the role of small molecules and metabolic systems as important mediators of metal resistance.

## Introduction

Pathogenic bacteria are subjected to several host antimicrobial effectors within the human body and must employ multiple mechanisms to resist these cellular stresses to survive, colonise and cause disease. Such stresses include antimicrobial peptides, reactive oxygen and nitrogen species, pH changes and nutrient availability which are reviewed in detail elsewhere [1]. Another important antimicrobial effector axis is the manipulation of metal ion availability during host–pathogen interactions [2]. Transition metal ions, including zinc (Zn), copper (Cu), iron (Fe) and manganese (Mn) are indispensable elements required for the correct function of numerous biological systems, functioning primarily as cofactors in catalytic sites of enzymes with important roles in a variety of processes, including electron and oxygen transport and detoxification of reactive species [3,4]. Metal ions can also be toxic due to their reactivity [5] and ability to displace other metals in catalytic sites of proteins [6–8].

The importance and toxicity of metals in biological functions makes it unsurprising that bacteria can sense and respond to changes in the availability of free metals in their external environment [9]. In turn, the host can respond to microbial infection by altering metal bioavailability either through sequestration, which can result in metal starvation, or by proactively mobilising and concentrating metals to intoxicate potential pathogens. Nutritional immunity is a form of host defence that exploits the relative bioavailability of trace elements to counteract infectious microbes and eliminate pathogens [2,10]. Metal homeostasis is the cellular management of metal levels to maintain bioavailability inside the cell whilst also minimising any damaging, cytotoxic effects of excessive metal build-up. This review primarily focusses on the mechanisms used by gram-positive bacteria to counteract metal toxicity in order to tolerate conditions of excess metal, such as those encountered following phagocytosis by macrophages or other immune cells [11,12]. We focus on the metals Cu and Zn due to their high reactivity as described in the Irving–Williams series [5] and their ability to form stable complexes with proteins that can disrupt function, either by displacing a preferred metal, leading to mismetallation [7,8], or by causing dysfunction by other means [13–18]. We also discuss resisting metal starvation and the ways through which bacterial pathogens subvert this important element of nutritional immunity.

## Transport-independent mediators of resistance to Cu intoxication

Although efflux systems are considered the primary means of metal ion detoxification [2,9,10,16,18–28], numerous transport-independent metal resistance effectors are reported to confer survival advantages during metal intoxication and these are described in detail below, including discussion of small molecules that directly affect metal availability, and those that subvert poisoning by metal intoxication.

### Glutathione buffering protects from Cu toxicity

**Figure 1.**
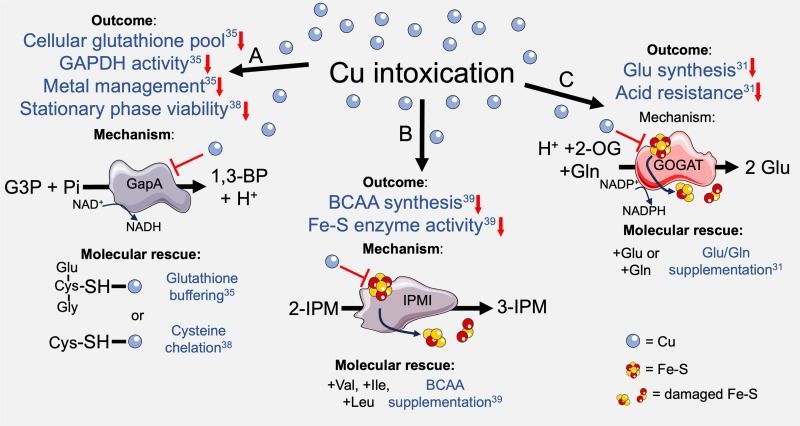
Cu intoxication in bacteria and molecular rescue by small molecules. (**A**) Cu intoxication causes a reduction (red arrows) in cellular glutathione, metal management [35] and reduction in viability at late stationary phase [38] in *S. pyogenes*. Cu-binding to histidine and cysteine residues in the catalytic site of GapA likely leads to a reduction in activity and subsequent flux through the fermentative pathway [35]. Growth inhibition in *S. pyogenes* undergoing Cu intoxication can be rescued by supplementation with the small molecules glutathione [35] or cysteine [38], likely due to chelation of excess Cu. (**B**) Cu binds to and destroys solvent-accessible Fe–S clusters in enzymes such as in IPMI and fumarase. This leads to growth inhibition of *E. coli* by a reduction in BCAA synthesis and reduced activity of multiple Fe–S enzymes [31,39], which can be partially restored by supplementing with BCAAs valine, leucine and isoleucine to bypass the BCAA synthesis block [39]. (**C**) Cu inactivates Fe–S cluster-containing GOGAT, impairing glutamate synthesis, which can be rescued by supplying exogenous glutamate or glutamine [31]. GAPDH/GapA, glyceraldehyde 3-phosphate dehydrogenase; G3P, glyceraldehyde-3-phosphate; Pi, inorganic phosphate; 1,3-BP, 1,3-bisphospho-d-glycerate; NAD^+^/NADH/NADP^+^/NADPH, nicotinamide adenine dinucleotide cofactors; SH, thiol group; 2-IPM, 2-isopropylmalate; 3-IPM, 3-isopropylmalate; IPMI, isopropylmalate isomerase; BCAA, branched-chain amino acid; GOGAT, glutamine oxoglutarate aminotransferase; 2-OG, 2-oxoglutarate; Cys, cysteine; Gly, glycine; Val, valine; Ile, isoleucine; Leu, leucine; Glu, glutamate; Gln, glutamine.

Glutathione is a small, non-protein, low molecular mass thiol synthesised from the amino acids glutamate, cysteine and glycine. Glutathione participates in numerous processes in bacteria including redox cycling, protection from oxidant damage [29], resistance to acid stress [30] and detoxification of Cu [29,31]. Glutathione complexes with Cu in aqueous solutions [32,33] and likely does so by assembling stable tetranuclear Cu_4_GS_6_ clusters, although the stoichiometry of Cu–glutathione complexes changes under different Cu concentrations [34]. Stewart et al. [35] demonstrate that glutathione also acts to buffer free Cu ions in *Streptococcus pyogenes*. Cu stress was studied in Δ*copA S. pyogenes* (defective for the primary Cu efflux system, CopA) and analysis of culture media demonstrates that a reduction in cellular glutathione levels was concurrent with the onset of Cu intoxication in cells growing at late-exponential phase. This was manifested in *S. pyogenes* by metabolic arrest due to reduction in both the consumption of glucose and production of lactic acid during fermentation, reduced activity of glyceraldehyde-3-phosphate dehydrogenase (GAPDH; EC 1.2.1.12: d-glyceraldehyde 3-phosphate + phosphate + NAD^+^ → 1,3-bisphospho-d-glycerate + NADH + H^+^; encoded by *gapA*), and dysregulation of Zn and Mn management, resulting in bacterial death [35]. The authors postulate that Cu-dependent growth inhibition in *S. pyogenes* likely occurs due to mismetallation of GapA (Figure 1A) and subsequent reduction in GAPDH activity, as was demonstrated in studies of Cu intoxication with *Staphylococcus aureus* GapA [36]. Supplementation assays showed that exogenous glutathione restored the growth of *S. pyogenes* in high Cu conditions, but the addition of other nutrients that were also limiting at late-exponential phase, including alanine/lysine, glycine/serine, isoleucine/leucine/valine, vitamins and nucleobases had no effect [35]. These authors conclude that cytoplasmic glutathione serves as an additional mode of metal intoxication resistance, likely through binding and chelating free Cu ions inside the cell, thus limiting the inhibition of core metabolic pathways.

### Amino acid supplementation subverts Cu intoxication

In another study of Cu intoxication in *S. pyogenes*, Dao et al. showed a reduction in viability during stationary phase in planktonic cultures undergoing Cu stress. The authors attributed this observation to nutritional deficiency since supplementation with a mixture of exogenous amino acids rescued this defect in survival at stationary phase. Supplementation with cysteine (Figure 1A), which forms Cu-binding ligands in proteins [37], enhanced survival in conditions of high Cu stress in *S. pyogenes* [38], consistent with prior studies of Cu intoxication in *Escherichia coli* [31]. Dao et al. [38] suggest that cysteine may rescue *S. pyogenes* from Cu toxicity due to this thiol-containing amino acid acting as a low-affinity pool for buffering free Cu. In comparing the work of Stewart et al. [35] and Dao et al. [38], which both analysed Δ*copA* mutants to study Cu intoxication in *S. pyogenes*, it is noteworthy that the two studies used distinctly different growth media. Such differences would affect the buffering capacity of small molecules in the medium for free Cu. This may explain the considerably higher concentration of Cu used by Dao et al. [38] (100–1000 μM) to induce Cu poisoning in Δ*copA S. pyogenes*, compared with Stewart et al. study (1–5 μM). Dao et al. [38] used Todd-Hewitt broth supplemented with yeast extract, a complex nutritionally rich media, replete with amino acids, whereas Stewart et al. [35] used a chemically defined medium based on RPMI, which would likely be replete with carbon sources but limiting in amino acid content. Striking differences in Cu stress phenotypes are reported in other studies of streptococci which have compared nutrient-rich and nutrient-limiting growth media of similar composition to the studies above [25], underscoring the crucial influence media composition has on measuring phenotypes relating to Cu intoxication.

### Branched-chain amino acids bypass Cu poisoning

In studies of *E. coli*, Macomber and Imlay [39] showed that Cu exerts toxicity by inducing branched-chain amino acid (BCAA) auxotrophy through poisoning of leucine synthesis pathways. In a series of experiments using wild-type (WT) and mutant *E. coli* defective for Cu export (*copA^−^ cueO^−^ cusCFBA^−^*) and a defined glucose medium, authors observed extreme sensitivity of *E. coli* to excess Cu, compared with similar assays performed using complex media [39]. Subsequent experiments showed Cu intoxication inactivated BCAA synthesis due to loss-of-function of dehydratase enzymes containing iron–sulfur (Fe–S) clusters such as isopropylmalate isomerase (IPMI; EC 4.2.1.33) involved in leucine biosynthesis. Supplementation with exogenous BCAAs (isoleucine, valine and leucine; Figure 1B) only partially restored growth during Cu intoxication [39], and consistent with this partial restoration, Macomber and Imlay [39] showed that growth-limiting processes linked to Cu poisoning occur outside of BCAA synthesis, likely due to direct damage of Fe–S clusters of other enzymes. For example, Cu poisoning resulted in the destruction of the Fe–S cluster of fumarase A (Figure 1B). The inhibitory effect of Cu on the purified fumarase A protein could be prevented by the addition of glutathione as a Cu-chelator, or enhanced, by the addition of histidine as a Cu-solubilisation agent, which likely prevented, or aided Cu delivery to the Fe–S cluster, respectively [39]. Importantly, work from the Imlay group also revealed that Cu toxicity proceeds in the absence of oxygen, suggesting that damage to Fe–S-containing enzymes such as IPMI and fumarase by Cu is unrelated to reactive oxygen species [39].

### Bypassing glutamate synthesis protects from Cu toxicity at low pH

In studies of Cu stress in *E. coli* exposed to acidic conditions, Djoko et al. [31] showed that excess Cu impaired glutamate biosynthesis through inactivation of glutamine oxoglutarate aminotransferase (GOGAT), which contains a solvent-exposed 4Fe–4S cluster. Loss of GOGAT function during Cu stress (Figure 1C) resulted in a reduction in the cellular glutamate pool [31]. Acidic conditions (in the absence of Cu) also triggered a reduction in cellular glutamate, likely due to consumption by glutamate decarboxylases (GadAB; H^+^ + glutamate → γ-aminobutyric acid + CO_2_) that support acid tolerance to maintain intracellular pH [31]. Interestingly, in *E. coli*, *ybaS* and *ybaT* are divergently transcribed from *copA* and expression of *ybaST* is up-regulated in response to excess Cu, although regulation of these genes is not directly associated with the Cu-sensing CueR [31]. The *ybaST* locus encodes a putative glutamine permease (YbaT) and a glutaminase (YbaS; glutamine + H_2_O → glutamate + NH_3_) that, together, comprise a system for glutamine-dependent acid resistance [31].

YbaS catalyses the breakdown of glutamine, forming glutamate and ammonia. Glutamate is subsequently decarboxylated by GadAB, consuming H^+^ and supporting acid stress resistance [40]. Djoko et al. [31] showed that high Cu stress could be subverted by supplementation with exogenous glutamine, and this rescue depends on functional YbaST. Glutamate supplementation also rescued growth during Cu intoxication, with this restoration likely due to a bypass of the Cu-impaired GOGAT enzyme [31]. Further studies by Djoko et al. showed that supplementation with other amino acids also subverted Cu stress in *E. coli*, including BCAAs, which partially restored growth at pH 7, consistent with the work of Macomber and Imlay [39]. Notably, though, restoration of growth by BCAAs did not occur at pH 5, suggesting there is a high requirement for glutamate/glutamine during acid tolerance under Cu stress [31]. Supplementation with asparagine and aspartate also subverted Cu stress in *E. coli* [31], consistent with their roles as substrates for an alternative pathway for synthesising glutamate via AspC and AnsAB that do not require Fe–S clusters as cofactors [31]. Exogenous glutathione and cysteine also conferred a survival advantage (Figure 1C), presumably due to buffering of Cu by these thiol compounds, but arginine did not protect *E. coli* from Cu in acidic conditions [31], despite its role as substrate for arginine deiminase (ADI), an alternative acid resistance pathway that is described in section ‘Ornithine supplementation disrupts Zn intoxication’.

### Histidine transport subverts Cu intoxication

**Figure 2.**
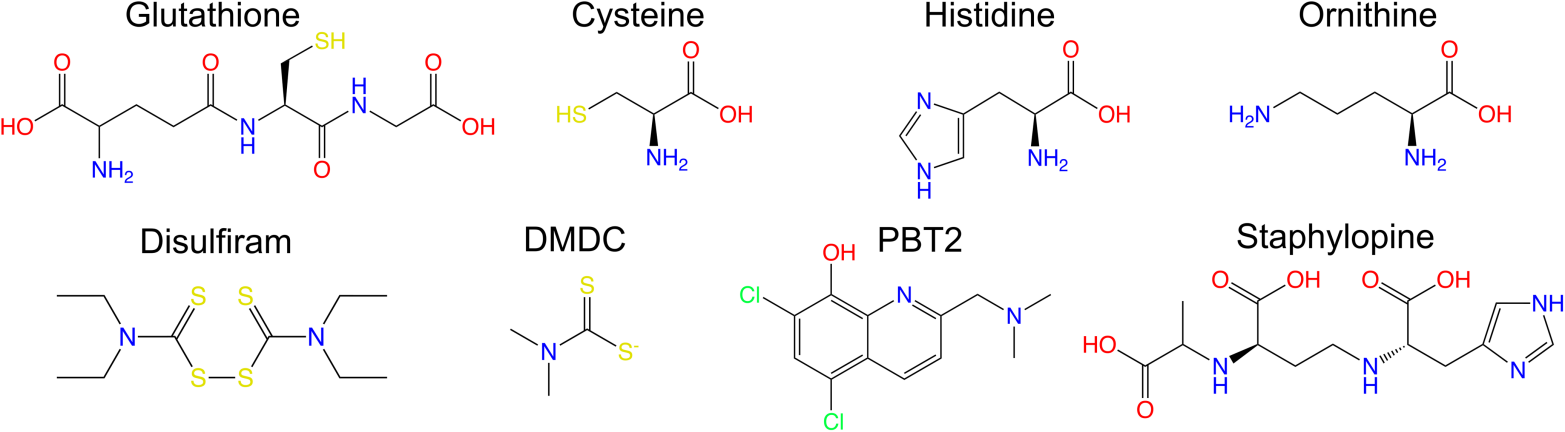
Chemical structures of small molecules that influence metal homeostasis. Certain molecules including glutathione, cysteine, histidine and ornithine can act to rescue bacteria from metal toxicity, whereas others can enhance metal toxicity, including disulfiram, *N,N*-dimethyldithiocarbamate (DMDC), PBT2 and staphylopine. ChEBI [99] structures: glutathione 16 856; cysteine 17 561; histidine 15 971; ornithine 15 729; disulfiram 4659; *N*′*N*′-dimethyldithiocarbamate 84 293; staphylopine 141 669.

Histidine is the only amino acid that contains an imidazole group (Figure 2) and has long been known for its ability to bind metals, including Zn and Cu ions, either within catalytic sites of proteins, or in solution [41,42]. Recently, a putative histidine ABC-type transporter was identified in a transposon screen of Cu intoxication as required for resistance to Cu stress in *Streptococcus agalactiae* [43]. A three-gene operon comprising *hisM* (encoding a permease), *hisJ* (ATP-binding protein) and *hisP* (substrate binding protein) was identified as part of the Cu-‘resistome’ by Goh et al. [43]. *S. agalactiae* is a histidine-auxotroph and must acquire this amino acid from the external environment directly, or by importing and degrading peptides containing histidine. HisMJP likely imports histidine, and analysis of a mutant defective for *hisMJP* revealed some interesting phenotypes. For example, in nutritionally replete Todd-Hewitt broth supplemented with high Cu, the Δ*hisMJP* mutant exhibited delayed entry into exponential phase, but no difference in stationary phase viability compared with WT [43]. Intracellular accumulation of Cu was significantly reduced in the Δ*hisMJP* strain compared with WT *S. agalactiae* during Cu intoxication, but the significance of this finding is unknown [43]. Goh et al. [43] suggest the extended lag phase in the Δ*hisMJP* strain could be explained by a period of metabolic reprogramming in order to obtain alternate sources of histidine, such as from peptides. In support of this theory, using a chemically defined medium devoid of peptides, the Δ*hisMJP* strain was hyper-sensitive to Cu stress [43]. Taken together, the work of Goh et al. suggests a novel role for the histidine transporter encoded by *hisMJP* in supporting Cu resistance. Interestingly, *in vitro* work by Macomber and Imlay [39] showed that histidine can act as a solubilising agent for Cu to promote interactions with fumarase A in an opposing fashion to the chelating activity of glutathione. Future work to decipher a mechanism for histidine transport in contributing to Cu resistance is now warranted.

## Transport-independent mediators of resistance to Zn intoxication

In addition to the mechanisms that subvert Cu poisoning described above that are independent from Cu efflux (section ‘Transport-independent mediators of resistance to Cu intoxication’), there are some factors involved in resisting Zn toxicity that are independent of Zn efflux and these are discussed below:

### Mn supplementation overcomes Zn intoxication

High concentrations of Zn can outcompete other metals, such as Mn, for binding sites in proteins [8,44], target exposed Fe–S clusters in enzymes [45] or disrupt Fe–S cluster biogenesis [46]. In *Streptococcus pneumoniae*, Zn displaces Mn in the solute-binding protein PsaA of the Mn-importing ABC-transporter PsaBCA [47]. In doing so, Zn-bound PsaA prevents internalisation, starving the cell of essential Mn [47] and leads to up-regulation of Mn-related genes including *psaBCA*. Mn is required for superoxide dismutase (SOD; SodA) and expression of *sodA* is down-regulated during Mn starvation, which can lead to susceptibility to oxidative stress [48]. Using assays incorporating Mn supplementation, McDevitt and colleagues showed that the ratio of Zn to Mn dictates the degree to which *S. pneumoniae* experiences Zn intoxication [47], and this is reflected *in vivo* in a mouse model of *S. pneumoniae* infection that exploits altered dietary Zn levels [14]. In the latter, Eijkelkamp et al. [14] show that although Mn levels do not change, Zn increases in the lungs following infection with *S. pneumoniae*, concurrent with up-regulation of genes associated with Zn intoxication (*czcD*) and Mn starvation (*psaA*). In other streptococci, it is likely that the Zn:Mn ratio is important for surviving Zn intoxication, since high Zn caused a significant reduction in total cellular Mn, and supplementation of Zn-intoxicated cells with exogenous Mn rescued growth under high Zn conditions [18,49].

### Ornithine supplementation disrupts Zn intoxication

Arginine catabolism was recently reported to be involved in Zn intoxication resistance in streptococci [18,50]. The ADI pathway converts arginine to ornithine and releases ATP and ammonia. The pathway commences with the ADI enzyme (encoded by *arcA*) and supports resistance to acid stress [51–53], biofilm formation and antibiotic tolerance [54] and host colonisation and virulence [55–59]. ADI of *S. pyogenes* is an anchorless, surface-displayed protein with potential as a vaccine antigen [60] and competes for the turnover of arginine in the host, thereby reducing host-production of the potent antimicrobial nitric oxide (NO) by the inducible NO synthase (iNOS) system [56]. Interestingly, the *arcABDC* locus in *S. agalactiae* was amongst the most strongly up-regulated transcripts in response to high Zn conditions [18]. Mutational analyses revealed a novel role for ADI in Zn homeostasis, since *arcA*-deficient *S. agalactiae* were significantly more sensitive to Zn intoxication than WT bacteria [18]. Supplementation assays showed that ornithine, the product of the ADI pathway, could rescue *S. agalactiae* from the toxicity of Zn, but notably, supplementation with arginine had no such effect [18]. This suggests that rescue of *S. agalactiae* is specific to ornithine, but the mechanism by which ornithine subverts Zn intoxication is yet to be elucidated.

In a separate study of Zn intoxication in *S. agalactiae*, the *arc* locus was recently highlighted in a transposon screen to identify members of the Zn-‘resistome’. Insertion-sequencing in high Zn conditions showed an over-representation of IS*S1* insertions in *arcR* and *argR* (∼10-fold enrichment), encoding putative regulators of the ADI pathway. Interestingly *arcA* (∼10-fold), *arcD* and *arcC* (∼2.5-fold) were also significantly over-represented in the Tn-sequencing dataset [50]. These data suggest that mutation in the putative *argR-arcR* and *arcABDC* operons is beneficial for surviving Zn intoxication, noting that IS*S1* insertion is likely polar on downstream or adjacent genes due to insertion of the entire pGh9:IS*S1* element. Consistent with the Tn-sequencing experiment, isogenic mutation in *arcR*, encoding a CRP-family repressor that likely regulates *arcABDC*, results in hyper-resistance to Zn stress [50]. Taken together, these observations collectively hint at a role for the *arcABDC* locus (encoding the ADI pathway) in supporting survival during Zn stress in streptococci and future work should seek to elucidate the mechanism involved.

## Small molecules that enhance the toxicity of Cu and Zn

Several compounds have been identified from studies of metal intoxication in bacteria, which work synergistically to significantly enhance the toxicity of metal ions towards bacterial pathogens. Some of these small molecules are emerging as promising antimicrobial agents and examples are described below and shown in Figure 2.

### Cu-bisthiosemicarbazones

Cu-bisthiosemicarbazones (Cu(btsc)) are lipophilic ionophore molecules that co-ordinate Cu(II) and were originally developed as anti-cancer therapeutics and for Alzheimer's disease [61]. Cu(btsc) compounds exhibit significantly enhanced toxicity towards *Neisseria gonorrhoeae*, compared with Cu-salts [62]. Cu(btsc) complexes are likely membrane permeable and enhance delivery of Cu into the cell, disrupting respiratory dehydrogenases [62,63]. Notably, though, susceptibility to Cu(btsc) complexes in bacteria depends on the organism's intrinsic efficiency of Cu efflux systems and/or reliance on Cu-sensitive, solvent-exposed Fe–S centres in core metabolic pathways [63].

### Disulfiram and dimethyldithiocarbamate

Other compounds that act synergistically with Cu to enhance bacterial killing include disulfiram [64] and *N*′*N*′-dimethyldithiocarbamate (DMDC). Disulfiram is an FDA-approved dithiol compound (Figure 2) that complexes with Cu and penetrates the cell envelope in a porin-independent manner. Disulfiram effectively killed *Mycobacterium tuberculosis* [64] and *S. aureus* [65] and does so by potentiating intracellular Cu stress but without increasing the intracellular concentrations of Cu, leading authors to suggest a model in which disulfiram protects Cu ions from the intracellular homeostatic mechanisms that would otherwise lead to Cu export [64]. DMDC is a related thiol that co-ordinates Cu and works synergistically to kill *S. pneumoniae* and *S. aureus* amongst other respiratory pathogens [66–68].

### PBT2

Another ionophore with potent antimicrobial activity is the hydroxyquinoline analogue PBT2, which facilitates the transport of metals such as Zn across biological membranes. PBT2 (Figure 2) has been shown to act synergistically with Zn to disrupt cellular homeostasis and enhance intracellular Zn concentrations in important drug-resistant pathogens including *S. pyogenes*, *S. aureus* and *Enterococcus faecalis* [69]. Strikingly, PBT2 also enhanced the efficacy of antibiotic treatment of these organisms, because combinatorial administration of antibiotics (erythromycin, methicillin or vancomycin) plus Zn and PBT2 re-sensitised these antibiotic-resistant bacteria and reduced the minimum bactericidal concentrations of the antibiotics that were required to kill the pathogen [69]. PBT2 was also shown to inhibit peptidoglycan synthesis and cell structure [70] through inactivation of *N*-acetylglucosamine-1-phosphate uridyltransferase (GlmU). PBT2 in combination with Zn and antibiotics has broad antimicrobial activity and can ‘break’ antibiotic resistance in both gram-negative and gram-positive pathogens [49,70–73]. Given the broad efficacy of PBT2 against a range of bacteria, and its safe-for-human use status [74–76], PBT2 is a promising candidate for combatting antimicrobial resistance in bacterial pathogens.

## Small molecules that enhance survival during metal starvation

Some small molecules can promote survival in conditions of metal starvation, such as when the host induces calprotectin-mediated metal sequestration [77], by enhancing the uptake of metals when at low concentrations. These are discussed in detail below and shown in Figure 2.

### Staphylopine is a scavenger of Zn

*Staphylococcus aureus* produces a range of small-molecule secondary metabolites that enable host colonisation during infection and provide a selective advantage over other microorganisms in nutrient-poor niches such as within a host. Among the secondary metabolites produced by *S. aureus*, staphylopine (StP) is a small molecule (Figure 2) broad-spectrum metallophore [78] that can chelate a range of divalent metals including Zn, Cu and Fe. StP is secreted by CntE and the metal-bound StP is recovered by CntABCDF [79]. Regulation of StP function via the *cnt* locus is tightly controlled by Zn (and to a lesser extent, Fe) at the transcriptional level [80] to enable the capture of Zn and promote resistance to Zn starvation [81]. Fine-tuning of StP production may also be mediated by activation or inhibition of CntM, which catalyses the final step in StP synthesis. CntM activity is highly sensitive to different metals and their concentrations; Zn and Cu are activators at low concentrations but completely inhibit CntM at high concentrations. Mn only activates CntM, and cobalt (Co) and nickel (Ni) are only inhibitors of CntM function [82], although metal selectivity of purified CntM *in vitro* may not reflect physiological function *in vivo*. Thus, control of StP synthesis is multifactorial and encompasses transcriptional and post-transcriptional signalling cues involving Fe, Cu and Zn. Interestingly, homologues of the genes required for StP biosynthesis are also found in *Yersinia pestis* [83] and *Pseudomonas aeruginosa* [84] with the analogous metallophore pseudopaline contributing to pathogenesis [85], suggesting a conserved strategy for metal acquisition during infection. Surprisingly, the synthesis of molecules like StP can be detrimental to the producing-bacteria, since the loss of StP efflux (CntE) attenuates growth and virulence [86], likely due to the accumulation of StP or a synthesis intermediate [87]. It is also noteworthy that StP can be a major driver of Cu intoxication in *S. aureus*. This metallophore usually functions to sequester Zn but can facilitate Cu uptake and lead to susceptibility in host niches with altered elemental abundances [88]. Although evidence supports an import role for StP in capturing Zn, it is now clear that the import of non-Zn metals by StP and the CntABCDF system can be toxic to *S. aureus*.

### Histidine catabolism protects from Zn starvation

Aside from a role for histidine in overcoming Cu intoxication described in section ‘Histidine transport subverts Cu intoxication’, histidine (Figure 2) also has a role in the subversion of host-induced Zn starvation. The gram-negative pathogen *Acinetobacter baumannii* exploits the properties of this amino acid in complexing free Zn ions to form histidine–Zn complexes (hereafter referred to as His–Zn). Nairn and colleagues show that the histidine utilisation (*hut*) genes [89], required for transport and catabolism of His–Zn into the cell, are up-regulated in response to calprotectin-induced Zn starvation [90]. During Zn-limitation, HutT imports His–Zn and histidine-ammonia lyase (HAL; encoded by *hutH*) catabolises histidine to yield urocanate and ammonia. Collectively, these products serve as carbon and nitrogen sources, and the pathway is essential for lung colonisation and pneumonia [91]. HAL-mediated destabilisation of the His–Zn complex also releases free intracellular Zn, and thus, His–Zn serves as a HAL-dependent source of labile Zn [90] that the bacterium can use to overcome host-induced Zn starvation.

## Conclusions and research gaps

The roles of metabolic processes and small molecules in contributing to metal resistance in bacteria is a rapidly emerging area and underscores a need for further development of a broader view of what constitutes a pathogen's metal ion ‘resistance repertoire’. There are many questions that highlight areas for further study and these will require multipronged approaches to make new discoveries. For example, recent work on *S. agalactiae* using a forward-facing transposon screen identified many new targets that contribute to resisting Cu intoxication [43], including *hisMJP* as mentioned above. None of the genes identified in the Tn-sequencing study (except *copA*) were detected as differentially expressed in a transcriptomic analysis of high Cu conditions of the same organism [25]; suggesting that certain metabolic processes within the cell (such as histidine transport), which are key to survival, may not themselves change in response to a given stress (in this instance, Cu intoxication). Further work is required to examine potential mechanisms involved, including a closer examination of metabolic pathways and secondary metabolites, or the role of small molecules in influencing bacterial survival during metal stress. Integrating a combination of transcriptomic, metabolomic, proteomic and genomic approaches (such as those used in [22,92–94]) will be important for making new fundamental discoveries. In addition, it will be interesting to learn why related pathogens with host-adapted lifestyles have retained or discarded certain metabolic pathways during the course of their evolution. For example, *S. pyogenes* contains a recognisable pathway for degrading histidine (via HAL), whereas *S. agalactiae* does not. It may be that discrete differences in what could be considered core metabolic pathways, contribute to the survival of different pathogens in niches within the body. Indeed, compared with *S. agalactiae* [25], it is surprising that *S. pyogenes* is far more sensitive to Cu [95]; both pathogens have been examined in studies of Cu intoxication, yet the latter seems unable to tolerate concentrations above 100 μM Cu in defined/standardized *in vitro* assays. Consistent with these observations, *S. agalactiae* has a bifunctional γ-glutamylcysteine synthetase-glutathione synthetase (GshAB) for glutathione synthesis [96], whereas *S. pyogenes* must rely solely on import [29]. The significance of glutathione synthesis versus uptake in the context of metal resistance should be explored further.

Metals such as Cu and Zn are only part of a mammalian host's arsenal of antimicrobial processes that immune cells use to destroy invading pathogens. What is unclear is precisely why some pathogens possess complex biosynthesis machinery to make certain small molecules (such as glutathione, histidine, arginine) whereas others must acquire them from the host. We propose there exists a molecular trade-off between the bioenergetic demands of biosynthesis pathways versus the likelihood of being ‘caught short’ in a host niche [97], where a particular metabolic pathway [98] can make the difference between survival and successful colonisation. Integrating metabolic, physiologic and pathogenesis studies are key challenges for the field ahead.

## Summary

- Efflux systems are well-studied effectors of metal resistance in bacteria.
- Cu intoxication inhibits function of numerous important enzymes by interactions with solvent-exposed Fe–S cofactors.
- Amino acid supplementation can subvert Cu toxicity, likely due to metabolic bypass as exemplified by BCAA synthesis.
- Glutathione as a small molecule supports bacterial survival during Cu intoxication by buffering of free Cu.
- Other small molecules can enhance an organism's ability to resist metal stress, including histidine, cysteine and ornithine, enabling growth in otherwise toxic concentrations of Cu or Zn.

## Open Access

Open access for this article was enabled by the participation of University of East Anglia in an all-inclusive *Read & Publish* agreement with Portland Press and the Biochemical Society under a transformative agreement with JISC.

## Abbreviations

ADI: arginine deiminase
BCAA: branched-chain amino acid
DMDC: *N*′*N*′-dimethyldithiocarbamate
GAPDH: glyceraldehyde-3-phosphate dehydrogenase
GOGAT: glutamine oxoglutarate aminotransferase
HAL: histidine-ammonia lyase
NO: nitric oxide
StP: staphylopine
WT: wild-type
